# Association between digestive diseases and sarcopenia among Chinese middle-aged and older adults: a prospective cohort study based on nationally representative survey

**DOI:** 10.3389/fnut.2023.1097860

**Published:** 2023-07-05

**Authors:** Guanghui Cui, Shaojie Li, Hui Ye, Yao Yang, Yingming Chu, Xiaofen Jia, Yue Feng, Miaomiao Lin, Xuezhi Zhang

**Affiliations:** ^1^Department of Integrated Traditional Chinese and Western Medicine, Peking University First Hospital, Beijing, China; ^2^Institute of Integrated Traditional Chinese and Western Medicine, Peking University, Beijing, China; ^3^School of Public Health, Peking University, Beijing, China; ^4^China Center for Health Development Studies, Peking University, Beijing, China

**Keywords:** digestive diseases, sarcopenia, middle-aged and older adults, China, CHARLS

## Abstract

**Objectives:**

Patients with digestive diseases frequently suffer from dyspepsia and malabsorption, which may lead to muscle loss due to malnutrition. However, it is not clear whether digestive diseases are associated with sarcopenia. This study aims to explore the longitudinal association between digestive diseases and sarcopenia in middle-aged and older adults based on a nationally representative survey from China.

**Methods:**

We used a prospective cohort study including 7,025 middle-aged and older adults aged ≥45 years from the 2011 to 2015 waves China Health and Retirement Longitudinal Study (CHARLS). Digestive diseases were identified using self-report. The assessment of sarcopenia was based on the Asian Working Group for Sarcopenia 2019 Consensus and included three components of muscle strength, physical performance, and muscle mass. Cox hazards regression was used to examine the association between digestive diseases and sarcopenia.

**Results:**

The prevalence of digestive diseases and the incidence of sarcopenia in middle-aged and older adults were 22.6% (95% CI = 21.6–23.6%) and 8.5% (95% CI = 7.8–9.1%). After adjusting for 15 covariates composed of three sets (demographic characteristics, lifestyles, and health status), digestive diseases were associated with a higher risk of sarcopenia (HR = 1.241, 95% CI = 1.034–1.490, *P* < 0.05). The associations were more pronounced among men, older adults aged 60–79, rural residents, and married people. In addition, the association between digestive diseases and sarcopenia was robust in the sensitivity analysis.

**Conclusion:**

Digestive diseases were associated with an increased risk of sarcopenia in middle-aged and older adults aged ≥45 years. Early intervention of digestive diseases may help to reduce the incidence of sarcopenia in middle-aged and older adults.

## 1. Introduction

Sarcopenia refers to a generalized and progressive skeletal muscle disorder including the loss of muscle strength and mass (1). A recent systematic review and meta-analysis indicated that the global prevalence of sarcopenia ranged from 10 to 27%, with the prevalence of severe sarcopenia varying between 2 and 9% (2). Sarcopenia not only decreased the quality of life for individuals but also caused serious economic and medical burdens to society and families. Numerous studies have shown that sarcopenia was associated with increased risks of adverse health outcomes such as falls, fractures (3), and mortality (4). In addition, it was identified as a predictor of increased health service use and healthcare costs according to the evidence from different countries (5, 6). Therefore, exploring its risk factors to achieve precise screening and early prevention has become the focus of interest in the field of clinical practice, geriatric and public health.

Previous studies mainly investigated the associated factors of sarcopenia from the perspective of sociodemographic characteristics (7) and nutritional status (8). In addition to these factors, studies have found that sarcopenia may also be secondary to some long-term diseases or co-exist with these pathological conditions (1, 9). Previous studies suggested an increased prevalence of sarcopenia in those with bone and joint diseases, cancer, chronic heart failure, chronic obstructive pulmonary disease, and diabetes (9). The long-term existence of these diseases would damage the physiological function of the body, lead to long-term chronic inflammation, and metabolic disturbances, and then induce sarcopenia (10). Based on these discovered factors, many studies have explored the intervention measures for sarcopenia, mainly involving exercise and nutritional supplements (11). A systematic review showed that exercise intervention can improve muscle strength and physical function, while the results of nutrition intervention were ambiguous (12), which may be related to the different kinds of nutrients taken. A recent systematic review and meta-analysis of nutrition supplement interventions found that different types of nutrients had different effects on the muscle mass and strength of older adults (13). In addition to the type of nutrients, the effect of nutrition supplements also depends on whether the individual can effectively absorb nutrients, which is directly related to the individual’s digestive and absorption function.

Patients with digestive diseases often have digestive disorders, which may lead to muscle loss due to poor absorption of nutrients. However, few studies have explored whether digestive diseases are associated with an increased risk of sarcopenia. A previous cross-sectional study that enrolled 303 patients with digestive diseases in Japan reported that the prevalence of sarcopenia was 32.0% (14), which indicated patients with digestive diseases often suffer from sarcopenia. However, it was impossible to explore the relationship between digestive diseases and sarcopenia, because this study only selected patients with digestive diseases as samples. Therefore, it is necessary to further examine the association between digestive diseases and sarcopenia to provide scientific evidence for the intervention of sarcopenia in the future. Malnutrition was identified as a highly prevalent complication in patients with digestive diseases such as gastroparesis (15), inflammatory bowel disease (16), pancreatic disease (17), cirrhosis (18), and non-alcoholic fatty liver disease (19). The Belgian population-based cohort study involving 534 community-dwelling older people showed that malnutrition was a strong predictor of the onset of sarcopenia (20). Recently, researchers put forward a view of the gut-muscle axis (21). They believed that the gastrointestinal tract and skeletal muscles interact with each other through hormones, gut microbes, and metabolites (22). This further implies the possible relationship between digestive diseases and sarcopenia.

Therefore, given the existing research gaps, we used a prospective cohort study to explore the association between digestive diseases and sarcopenia. This will provide evidence based on the Chinese community population level for further examining the association between digestive diseases and sarcopenia. It is contributed to simply targeting high risk populations in community screening and intervention of sarcopenia.

## 2. Materials and methods

### 2.1. Participants

**FIGURE 1:**
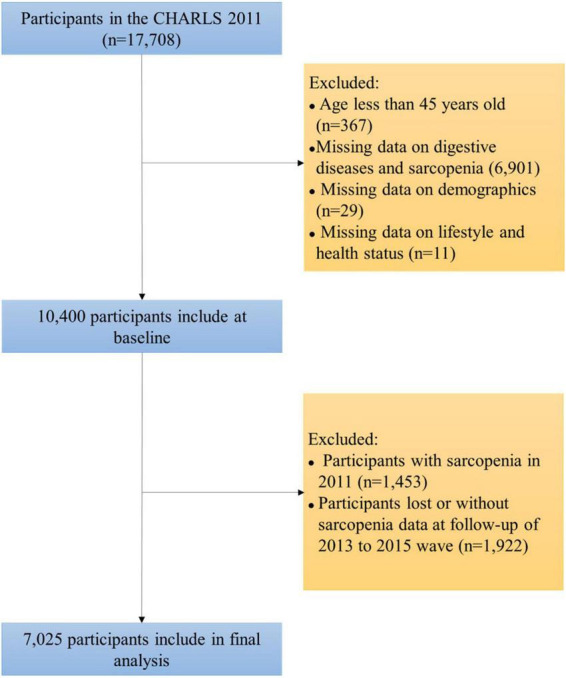
The selection process of participants.

We used the 2011 to 2015 waves of China Health and Retirement Longitudinal Study (CHARLS) data to conduct a prospective cohort study. CAHRLS is a national survey of middle-aged and older adults in China, which adopts a multi-stage stratified probability sampling method and has good national representation (23). CHARLS started the baseline survey in 2011, followed by a follow-up about every 2 years. At present, the survey data for four times in 2011, 2013, 2015, and 2018 have been disclosed. Since the data in 2018 only published questionnaire data, but not biomarker data, we used the data in 2011–2015 to explore the association between digestive system diseases and sarcopenia. In 2011, CHARLS recruited 17,708 participants. After excluding samples under the age of 45 and those with missing data, 10,400 participants were included at baseline. Subsequently, we excluded participants with sarcopenia in 2011 and those who were lost or missing sarcopenia data in the 2013 and 2015 surveys, and finally 7,025 participants entered the final analysis. The sample selection process is shown in Figure 1. CHARLS has been approved by the Ethics Committee of Peking University (No. IRB00001052-11015), and all participants have obtained informed consent.

### 2.2. Measures

#### 2.2.1. Digestive disease

Digestive diseases were identified using self-report. Participants were asked, “Have you been diagnosed with Stomach or other digestive diseases (except for tumor or cancer) by a doctor?” When participants answered “yes” was determined to have digestive diseases. In this study, we defined digestive diseases as a binary variable (no vs. yes).

#### 2.2.2. Sarcopenia

The assessment of sarcopenia was based on the Asian Working Group for Sarcopenia 2019 Consensus and includes three components of muscle strength, physical performance, and muscle mass (24). Muscle strength was measured using a standardized handgrip strength meter (Yuejian WL-1000, China). The handgrip strength of men below 28 kg and women below 18 kg is considered low muscle strength (24). Five-time chair stand tests were used to evaluate the individual’s physical performance. The time of 5-time chair stand tests ≥12 s is considered low physical performance (24). The muscle mass was evaluated by the appendicular skeletal muscle mass (ASM). Since CHARLS does not use instruments to measure ASM, we used previously validated anthropometric formulas (ASM = 0.193 × body weight + 0.107 × height − 4.157 × sex (men = 1, women = 2) − 0.037 × age − 2.631) to estimate ASM (25). To reduce the potential impact of height on ASM, we adjust the height of ASM by dividing ASM by the square of height (ASM/Ht2). Referring to the previous study (26), we set the low muscle mass as lower than the 20th percentile of ASM for sex. Low muscle mass + low muscle strength or low physical performance was considered sarcopenia.

#### 2.2.3. Covariates

Covariates in this study include three sets: demographic characteristics, lifestyles, and health status. Demographic characteristics included age, sex, residence (rural or urban), marital status (married or unmarried), and education level (illiterate, or primary school, or middle school and above). Lifestyles included smoking (current non-smoker or current smoker), drinking (current non-drinker or current drinker), exercise (hardly or regularly), social activity participation (No or Yes), and meal frequency (<3 or ≥3 meals per day). Health status included chronic diseases (No or Yes), cognitive function [cognitive normal or mild cognitive impairment (MCI)], functional limitations (No or Yes), visual impairment (No or Yes), and hearing impairment (No or Yes). More details of the measurement of covariates are in the Supplementary material.

### 2.3. Statistical methods

STATA version 17.0 (StataCorp, College Station, TX, USA) was used to perform all statistical analyses. We used the Chi-square test to compare the differences in demographic characteristics, lifestyles, and health status between groups with and without digestive diseases. Cramer’s V statistic was used to estimate the effect size. Cox hazards regression was used to examine the association between digestive diseases and sarcopenia. Hazard Ratio (HR) and 95% confidence interval (CI) were used to estimate the strength of association and statistical significance, respectively. We estimated the following four models: model 1 was unadjusted; model 2 adjusted age, sex, residence, marital status, and education level; model 3 adjusted smoking, drinking, exercise, social activity participation, and meal frequency based on model 2; model 4 further adjusted chronic diseases, cognitive function, functional limitations, hearing impairment, and visual impairment based on model 3. To test the heterogeneity of the results, we conducted subgroup analyses among sex, age, residence, and marital status. Meanwhile, we also examined the interaction of digestive diseases with sex, age, residence, and marital status for associations with sarcopenia. In addition, we excluded individuals with MCI for sensitivity analysis to test the robustness of the association between digestive diseases and sarcopenia. We set *P* < 0.05 as statistically significant.

## 3. Results

### 3.1. Descriptive statistics

The mean age of 7,025 participants was 57.3 years (SD: 9.4). The prevalence of digestive diseases and the incidence of sarcopenia in middle-aged and older adults were 22.6% (95% CI = 21.6–23.6%) and 8.5% (95% CI = 7.8–9.1%). More detailed information regarding the characteristics of participants according to digestive diseases is shown in Table 1.

**TABLE 1:** Characteristics of participants according to digestive diseases.

Variables	Total sample	No digestive diseases	Digestive diseases	Effect size	*P*-value
Total sample, *n* (%)	7,025 (100.0)	5,440 (77.4)	1,585 (22.6)		
Age, yeas				0.007	0.834
45–59	4,453 (63.4)	3,445 (63.3)	1,008 (63.6)		
60–79	2,509 (35.7)	1,948 (35.8)	561 (35.4)		
≥80	63 (0.9)	47 (0.9)	16 (1.0)		
Sex, *n* (%)				0.031	0.009
Male	3,539 (50.4)	2,786 (51.2)	753 (47.5)		
Female	3,486 (49.6)	2,654 (48.8)	832 (52.5)		
Residence, *n* (%)				0.039	0.001
Rural	5,715 (81.4)	4,381 (80.5)	1,334 (84.2)		
Urban	1,310 (18.6)	1,059 (19.5)	251 (15.8)		
Marital status, *n* (%)				0.023	0.052
Married	6,356 (90.5)	4,902 (90.1)	1,454 (91.7)		
Unmarried	669 (9.5)	538 (9.9)	131 (8.3)		
Educational level, *n* (%)				0.060	<0.001
Illiterate	1,563 (22.2)	1,193 (21.9)	370 (23.3)		
Primary school	2,928 (41.7)	2,201 (40.5)	727 (45.9)		
Middle school and above	2,534 (36.1)	2,046 (37.6)	488 (30.8)		
Smoking status, *n* (%)				0.006	0.596
Current non-smoker	4,139 (58.9)	3,196 (58.8)	943 (59.5)		
Current smoker	2,886 (41.1)	2,244 (41.3)	642 (40.5)		
Drinking status, *n* (%)				0.031	0.010
Current non-drinker	4,485 (63.8)	3,430 (63.1)	1,055 (66.6)		
Current drinker	2,540 (36.2)	2,010 (36.9)	530 (33.4)		
Exercise, *n* (%)				0.013	0.286
Hardly	4,031 (57.4)	3,140 (57.7)	891 (56.2)		
Regularly	2,994 (42.6)	2,300 (42.3)	694 (43.8)		
Social activity participation, *n* (%)				0.020	0.094
No	3,367 (47.9)	2,578 (47.4)	789 (49.8)		
Yes	3,658 (52.1)	2,862 (52.6)	796 (50.2)		
Meal frequency, *n* (%)				0.006	0.617
<3 meals per day	861 (12.3)	661 (12.2)	200 (12.6)		
≥3 meals per day	6,164 (87.7)	4,779 (87.8)	1,385 (87.4)		
Chronic disease, *n* (%)				0.131	<0.001
Without	2,918 (41.5)	2,449 (45.0)	469 (29.6)		
≥1	4,107 (58.5)	2,991 (55.0)	1,116 (70.4)		
Cognitive function, *n* (%)				0.023	0.053
CN	6,018 (85.7)	4,684 (86.1)	1,334 (84.2)		
MCI	1,007 (14.3)	756 (13.9)	251 (15.8)		
Functional limitations, *n* (%)				0.097	<0.001
No	5,553 (79.1)	4,416 (81.2)	1,137 (71.7)		
Yes	1,472 (20.9)	1,024 (18.8)	448 (28.3)		
Hearing impairment, *n* (%)				0.079	<0.001
No	3,291 (46.8)	2,664 (49.0)	627 (39.6)		
Yes	3,734 (53.2)	2,776 (51.0)	958 (60.4)		
Visual impairment, *n* (%)				0.070	<0.001
No	1,701 (24.2)	1,405 (25.8)	296 (18.7)		
Yes	5,,324 (75.8)	4,035 (74.2)	1,289 (81.2)		
Sarcopenia, *n* (%)				0.035	0.003
No	6,430 (91.5)	5,008 (92.1)	1,422 (89.7)		
Yes	595 (8.5)	432 (7.9)	163 (10.3)		

CN, cognitively normal; MCI, mild cognitive impairment.

### 3.2. Cox hazards regression

Table 2 displays the results of the association between digestive diseases and sarcopenia among the whole sample. Model 1 showed that digestive diseases were significantly associated with a high risk of sarcopenia (HR = 1.304, 95% CI = 1.089–1.562, *P* < 0.01). After adjusting for age, sex, residence, marital status, and educational level in model 2, the association weakened but was still significant (HR = 1.270, 95% CI = 1.061–1.522, *P* < 0.01). And then, we further adjusted smoking, drinking, exercise, social activity participation, and meal frequency based on model 2, digestive diseases were also associated with a higher risk of sarcopenia (HR = 1.266, 95% CI = 1.057–1.516, *P* < 0.05) in model 3. Finally, model 4 shows that digestive diseases still increased the risk of sarcopenia (HR = 1.241, 95% CI = 1.034–1.490, *P* < 0.05) after adjusting health status.

**TABLE 2:** Associations between digestive diseases and sarcopenia among whole sample.

Model	HR	SE	95% CI	*P*-value
Model 1	1.304	0.120	1.089–1.562	0.004
Model 2	1.270	0.117	1.061–1.522	0.009
Model 3	1.266	0.117	1.057–1.516	0.011
Model 4	1.241	0.006	1.034–1.490	0.021

Model 1 was unadjusted; model 2 adjusted age, sex, residence, marital status, and education level; model 3 further adjusted smoking, drinking, exercise, social activity participation, and meal frequency based on model 2; model 4 further adjusted chronic diseases, cognitive function, functional limitations, hearing impairment, and visual impairment based on model 3.

### 3.3. Subgroup analysis

**FIGURE 2:**
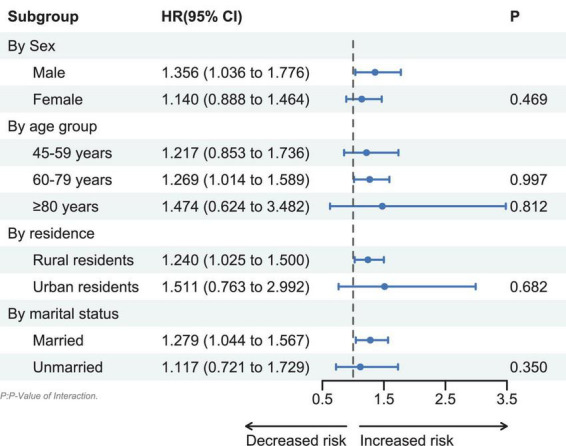
Subgroup analyses of associations between digestive diseases and sarcopenia.

The results of the subgroup analysis showed that the association between digestive diseases and sarcopenia was heterogeneous among sex, age, residence, and marital status. Specifically, the association was more pronounced among males, older adults aged 60–79 years, rural residents, and married people. However, we found no significant interaction effects. Details of the subgroup analysis are shown in Figure 2.

### 3.4. Sensitivity analysis

After excluding participants with MCI, digestive diseases were still associated with sarcopenia (HR = 1.387, 95% CI = 1.126–1.707, *P* = 0.002). This result suggested that the association of digestive diseases with sarcopenia was robust.

## 4. Discussion

Based on a nationally representative survey, this study explored the longitudinal association between digestive diseases and sarcopenia in middle-aged and older adults in China. As far as we know, this is the first study to explore the above association based on the Chinese population. This study found that digestive diseases were associated with a higher risk of sarcopenia among Chinese middle-aged and older adults, which further enriched the relevant factors of sarcopenia, and provided new intervenable factors for future prevention and intervention.

Although previous studies have not directly explored the above associations, some studies have investigated the association between gastrointestinal diseases and the components of sarcopenia. A longitudinal study based on UK Biobank showed that gastrointestinal diseases predicted a decline in grip strength after 9 years of follow-up (27). This further supported our findings and provided evidence from observational studies for the theory of the gut-muscle axis. It should be noted that our study excluded digestive system cancer from digestive diseases, because cachexia and sarcopenia are very common complications of cancer patients (28), which may introduce the potential bias. This study also found that the association between digestive diseases and sarcopenia was different in sex, age, residence, and marital status. This association only applied to males, older adults aged 60–79 years, rural residents, and married people. To begin with, it is found in males, rural residents, and married people that there were significant associations between digestive diseases and sarcopenia, which may be related to lower health services utilization. The previous national survey of older adults in China found that males, rural residents, and married people were less likely to use outpatient and inpatient services than females, urban residents, and the unmarried (29), which may make them unable to get timely treatment when they were ill, thus increasing the harm of digestive diseases to health. Secondly, the association between digestive diseases and sarcopenia was found only in participants aged 60–79 years, which may be due to the lower incidence of sarcopenia in middle-aged people aged 45–59 and the lower number of the oldest-old over 80 years old. In statistical analysis, a low incidence rate and sample size may lead to insufficient statistical power. However, since no significant interactions were found, the differences in these subgroup analyses may be due to a reduction in sample size after grouping. In the future, larger sample studies are needed to analyze the results of different subgroups.

The mechanism of association between digestive diseases and an increased risk of sarcopenia may involve an imbalance of gut microbiota, malnutrition, and long-term inflammatory condition. Firstly, most digestive diseases have changes in gut microbiota, which may play a role in the pathophysiological process of muscle mass decline. The gut microbiota of patients with chronic liver disease showed a change of decreasing abundance of Firmicutes (Ruminococcaceae and Lachnospiraceae), Prevotellaceae, and an increase of Enterobacteriaceae, Proteobacteria (30). The gut microbiota of patients with IBD showed a change of decreasing abundance of Firmicutes (*Eubacterium*, *Christensenellaceae*, and *Faecalibacterium prausnitzii*), and an increase of *Actinomyces*, *Escherichia coli* (31). Moreover, other digestive diseases including *Helicobacter pylori* infection were also associated with alterations in the gut microbiome (32, 33). Researchers analyzed the gut microbiome of subjects with sarcopenia or low skeletal muscle mass, and the results indicated that the abundance of Firmicutes (*Eubacterium*, Ruminococcaceae, Lachnospiraceae, *F. prausnitzii*) decreased (34, 35). These changes were consistent with the characteristics of gut microbes in patients with liver and gastrointestinal diseases to a certain extent. The microbes decreased in digestive diseases may produce some metabolites such as short-chain fatty acids (SCFAs), secondary bile acids (BAs), and some amino acids. They promoted skeletal muscle health by activating G protein-coupled receptors (GPR41/13/43) and inhibiting HDACs to further regulate the signal pathways related to insulin resistance, inflammatory response, and oxidative stress (36).

Secondly, patients with digestive diseases often face the risk of malnutrition. It was reported that 32.0–61.5% of patients with various digestive diseases suffer from malnutrition (17, 37, 38). Anorexia and abdominal discomfort are the main symptoms of patients with digestive diseases, which may lead to a decrease in their dietary intake and mean a lack of raw materials for muscle protein synthesis. In addition, various pathological changes associated with digestive diseases, such as digestive enzyme deficiency, decreased synthesis or secretion of conjugated BAs, reduced intestinal absorptive area, and dysbacteriosis can cause maldigestion and malabsorption, making nutrients unable to be used for skeletal muscle metabolism (39). In addition, the association between digestive diseases and sarcopenia may also be related to the reduction of nutrient absorption caused by taking medicine in patients with digestive diseases. Proton pump inhibitors (PPIs) have become the first choice for the treatment of acid-related digestive diseases, thus the use of PPI has been increasing in recent decades (40, 41). A large number of studies have found that the use of PPI was related to the reduced absorption of micronutrients such as vitamins C, D, and magnesium (42, 43). Meanwhile, more and more studies have shown that low micronutrient intake was associated with an increased risk of sarcopenia (44).

Thirdly, chronic low-grade inflammation is an important pathological mechanism for the development of sarcopenia. Gastritis, pancreatitis, chronic liver disease, inflammatory bowel disease, irritable bowel syndrome, and other digestive diseases infected were often accompanied by inflammation and caused elevated levels of proinflammatory cytokines such as tumor necrosis factor-alpha (TNF-α), interleukin 6 (IL-6), and myostatin (22, 45, 46). TNF-α, IL-6, and myostatin may activate the expression of the muscle-specific E3 ligases muscle RING-finger protein-1 (MuRF-1) and muscle atrophy F-Box (MAFbx) through regulating the p38 mitogen-activated protein kinase (MAPK) pathway and the nuclear factor-kappa B (NF-κB) pathway (47). MuRF-1 and MAFbx can further activate the ubiquitin proteasomal system (UPS) to promote protein degradation or proteolysis of skeletal muscle (47). The abnormal autophagy enhanced by phosphoinositide 3-kinase (PI3K)/protein kinase B (AKT)/mammalian target of rapamycin (mTOR) pathway was also associated with the process of muscle atrophy mediated by inflammation (48). These mechanisms provide a possible explanation for the relationship between digestive system diseases and sarcopenia. This also suggests that it is necessary to include intestinal flora or inflammatory factors in future research to explore whether these biomarkers can mediate the relationship between digestive diseases and sarcopenia.

It is worth noting that our study has several advantages. First of all, based on a nationally representative data, we explored the association between digestive diseases and sarcopenia using a prospective cohort study for the first time, which has good extrapolation and a higher level of evidence. Secondly, we controlled a set of covariates such as the social demographic characteristics, health conditions, and lifestyles to make the research results more stable. In particular, we have controlled the prevalence of chronic diseases, and the results showed that digestive diseases still significantly increased the risk of sarcopenia, which indicates that digestive diseases were significantly correlated with sarcopenia independently of other chronic diseases. In addition, we also conducted a subgroup analysis, which will help to find vulnerable groups more easily in the early prevention of sarcopenia in the future. Finally, we also conducted a sensitivity analysis, and the results suggested that the association between digestive diseases and sarcopenia was robust. The results of this study provide new ideas for early screening and prevention of sarcopenia in middle-aged and older people in community settings in the future. Community health workers can predict the incidence of sarcopenia 4 years later by asking middle-aged and older people if they have digestive diseases. Considering the availability of self-reported digestive diseases, this study has significant public health implications for reducing the burden of sarcopenia.

In addition, our study has some limitations. The cohort study cannot infer the causal relationship between digestive diseases and sarcopenia, which is our first limitation. In the future, causal inference methods are needed to enhance the causal effect of the above results. Second, we used self-reported digestive diseases instead of being diagnosed by doctors themselves, which may lead to evaluation bias. Although the survey has emphasized the need for participants to report diseases based on previous doctors’ diagnoses, it is undeniable that there may be bias. Due to the data limitations of CHARLS, previous studies (49, 50) have also used digestive diseases assessment methods consistent with this study, and have emphasized the limitations of self-reported bias and measurement bias. However, on the other side, given the scarcity of primary medical resources, if the risk of sarcopenia in middle-aged and older adults after 4 years can be determined based on self-reported digestive diseases, this may be of great value for early intervention and prevention of sarcopenia, as self-reported digestive diseases are very easy to obtain and do not require accurate medical equipment diagnosis. In addition, we have not collected subtypes of digestive diseases, such as gastritis, gastric ulcer, and inflammatory bowel disease. It is necessary to further explore the relationship between specific digestive diseases and sarcopenia in the future. Moreover, sarcopenia may be closely related to the diet of participants, however, since the CHARLS did not involve specific dietary assessments, we only included daily dietary frequency as a covariate, which may result in confounding bias. Finally, we excluded some missing data in the process of sample selection, which may introduce selection bias. However, due to the large number of samples included in our study, the impact of selection bias may be small.

## 5. Conclusion

Digestive diseases were associated with an increased risk of sarcopenia in middle-aged and older adults aged ≥45 years. Early intervention of digestive diseases may help to reduce the incidence of sarcopenia in middle-aged and older adults.

## Data availability statement

The datasets presented in this study can be found in online repositories. The names of the repository/repositories and accession number(s) can be found below: the data used in this study can be obtained from CHARLS official website (https://charls.pku.edu.cn/en/).

## Ethics statement

The studies involving human participants were reviewed and approved by the Ethics Committee of Peking University. The patients/participants provided their written informed consent to participate in this study.

## Author contributions

GC, SL, and XZ: conceptualization. GC and SL: methodology, validation, and formal analysis. GC: software and writing—original draft preparation. HY, GC, SL, and XZ: writing—review and editing. XZ: supervision. YY, YF, YC, XJ, and ML: project administration. All authors had read and agreed to the published version of the manuscript.
