# Supplementary material for: Association between digestive diseases and sarcopenia among Chinese middle-aged and older adults: a prospective cohort study based on nationally representative survey
Source: Front Nutr. 2023 Jul 5;10:1097860. doi: 10.3389/fnut.2023.1097860 (PMC10354238; doi:10.3389/fnut.2023.1097860)
Supplement: Supplementary file 1 [file Data_Sheet_1.docx]

**Supplementary material**

**The measurement of covariates**

The cognitive function was assessed using the validated episodic memory test and the Telephone Interview of Cognitive Status (TICS)^1^. The episodic memory test included immediate word recall (0–10 point) and delayed word recall (0–10 point). TICS included orientation (0–5 points), visuo-construction (0–1 point) and attention (0–5 points). The sum of episodic memory test and TICS scores is the total score of cognitive function, ranging from 0 to 31. According to the previous scoring criteria^2^, we divided all participants into 11 age groups based on an age group of 5 years old. MCI was determined when the cognitive function score of each group was lower than the mean minus one standard deviation (SD). Functional limitations were evaluated using 6 items (dressing, bathing, feeding, moving from bed to chair, using the toilet, and maintaining continence) of activities of daily living (ADLs) and 5 items (doing housework, cooking, shopping, managing money, and taking medication) of instrumental ADLs ^3^. Participants were asked if they had difficulty completing above 11 activities? The answer "difficult and unable to complete" was determined as functional limitations. Hearing and visual impairment were evaluated using self-reported 5-category hearing and visual status assessments. The answers "excellent, very good, and good" were considered to have no hearing or visual impairment, and the answers "fair, poor" were considered to have hearing or visual impairment.

**Reference**

1. Yao Y, Wang K, Xiang H. Association between cognitive function and ambient particulate matters in middle-aged and elderly Chinese adults: Evidence from the China Health and Retirement Longitudinal Study (CHARLS). Sci Total Environ. 2022;828:154297. doi:10.1016/j.scitotenv.2022.154297.

2. Hu Y, Peng W, Ren R, Wang Y, Wang G. Sarcopenia and mild cognitive impairment among elderly adults: The first longitudinal evidence from CHARLS. J Cachexia Sarcopenia Muscle. 2022;13(6):2944-2952. doi:10.1002/jcsm.13081.

3. Gong J, Wang G, Wang Y, et al. Nowcasting and forecasting the care needs of the older population in China: analysis of data from the China Health and Retirement Longitudinal Study (CHARLS) [published correction appears in Lancet Public Health. 2023 Feb;8(2):e98]. Lancet Public Health. 2022;7(12):e1005-e1013. doi:10.1016/S2468-2667(22)00203-1.
